# “Following the Breath:” A Trauma-Informed Intervention for Educator Wellness in Rural Montana

**DOI:** 10.3390/educsci13010023

**Published:** 2022-12-26

**Authors:** Lauren Davis, Alexandra Aylward, Brandon G. Scott, Jonathan Jacobs

**Affiliations:** 1 Montana State University, College of Education, Health, and Human Development and Department of Psychology, Bozeman, MT 59717, USA; 2 University of Texas-Austin, College of Education, Austin, TX 78712, USA

**Keywords:** trauma, teachers, rural education, stress, burnout, mental health, intervention

## Abstract

Given the prevalence of mental health issues for both educators and adolescents in rural Montana, this project is designed to help mitigate the impact of stressors by providing coping strategies linked to improvements in overall mental health outcomes for teachers, which may ultimately lead to improved co-regulation of students and classroom climate. The immediate goal of this pilot study was to measure physical and mental health outcomes of educators resulting from a remotely-delivered trauma-informed yoga intervention. Findings suggest improvements in participants’ depression and anxiety levels, trauma symptoms, sleep quality, and non-significant changes in heart rate variability and cortisol levels.

## 1. Introduction

“I’m no fun anymore…. I’m not connected with my class anymore. I’m not connected to ANYONE in my building. I’m so [expletive] lonely!!!” [1] These words are indicative of the frames of mind of not only some of the participants in the following study, but among many educators nationally during the ongoing COVID-19 pandemic.

America is experiencing an unprecedented mental health emergency; in a survey sampling over 1.5 million Americans in September 2020, more than 8 out of 10 who took a depression screen scored as having symptoms of moderate to severe [2]. Specifically, our nation’s educators’ mental health has plummeted. Another survey recently given to educators cites that more than a quarter of teachers and principals report depression symptoms, and nearly 75% of teachers and 85% of principals point to high levels of job-related stress [3]. Given the number of educators leaving the profession recently, along with their cited reasons for resignations (burnout, lack of support, etc.), it is critical to support educator mental health in order to concurrently bolster student well-being [4,5]. By providing coping mechanisms for educators in the workforce through a trauma-informed yoga intervention, the aim of this study was to create opportunities for improvement of mental health outcomes, along with improved career satisfaction and reduced burnout for teachers.

## 2. Review of the Literature

### 2.1. Context and Theoretical Framework (CBPR)

Montana is home to high rates of deleterious mental health issues; according to the United Health Foundation, Montana ranked nationally as the third highest in suicide rates in 2021 [6]. Per every 100,000 residents, Montana has a rate of suicide of 27.0 people, whereas the average suicide rate for the nation is 13.5 per 100,000 residents [6,7]. Tragically, these numbers have increased over the last few years; for example, from 2017 to 2018 the rate of suicides per 100,000 people in Montana was 26.2 [7]. Relatedly, rates of depression in Montana for adults were reportedly 22.6% in 2022, whereas the national rate of adults who reported depression in 2020 was 19.5% [6]. Thus, it is imperative to find ways to mitigate the ongoing mental health and suicide crisis in Montana.

In order to best meet the unique and varying needs of rural communities in Montana, this research design drew upon elements of community-based participatory research (CBPR) [8,9]. CBPR offers an orientation, rather than a set of methods, to provide insight to varying perspectives, particularly within marginalized contexts. Within educational research, CBPR often shifts the focus from the academic researcher to the community and schools. Therefore, CBPR “recognizes the importance of involving members of a study population as active and equal participants, in all phases of the research project, if the research process is to be a means of facilitating change” [10]. This project directly addressed needs identified by rural community members, school district leadership, mental health professionals, and educators themselves.

### 2.2. Educator Wellness

Montana also finds itself struggling with teacher retention. 61% of Montana schools are classified as rural-remote schools, and these schools have historically higher rates of teacher turnover when compared to their urban counterparts [11]. Specifically, in 2019, urban schools’ turnover rates were 21%, whereas 29% of teachers in rural-remote schools subsequently resigned [11]. In Montana, 2019 studies indicated that nearly 75% of districts in Montana are designated as rural, and 65% of those districts reported difficulties in filling teacher vacancies [12,13]. The school district in which this study took place is currently classified as a rural-remote school; thus, this study site indicates a need for promoting teacher retention, as it currently has a high turnover rate.

Related to educator turnover, teachers have one of the highest burnout rates of any profession [14]. Indeed, teacher burnout is as high as professions such as health care workers [15]. However, not all burnout is equal. Burnout for teachers is highest in rural areas [16] for male teachers, and teachers of older students [17]. Burnout is due, in part, to high stress and increased mental health difficulties [14,16,18]. These two issues have been exacerbated by the COVID-19 pandemic [19,20]. According to a 2022 survey disseminated by the National Education Association, more than 50% of respondents plan to leave the profession “sooner than planned” due to job-related stressors [19]. It has been shown, however, that an increase in protective factors, such as lower stress, helps reduce teacher burnout [18,21]. Additional protective factors include increased community connectivity and out-of-work activities [21].

In order to increase these protective factors, our study sought to address both educator stress as well as burnout. Yoga has been shown to reduce stress and simultaneously improve stress management [22]. Further, yoga, when paired with mindfulness activities, has also been shown to reduce stress, specifically during the early months of the COVID-19 pandemic [15]. An additional benefit of mindfulness activities is an increase in resilience to stress [23]. Moreover, educators have also exhibited better sleep schedules and less rumination about work at home while maintaining a yoga practice [24]. From this, our study examined both validated survey measures, as well as biological markers, to measure the impact of a yoga and mindfulness intervention on educator wellbeing and job satisfaction.

## 3. Intervention Design: Trauma Informed Yoga

Yoga focuses on helping individuals move from the sympathetic nervous system to the parasympathetic nervous system [25]. During the practice, yoga practitioners are required to focus on breathing and existing in the moment; as a result, those who practice yoga exhibit fewer trauma response symptoms (i.e., being locked in fight-or-flight mode) and report more relaxation [25]. Trauma-informed yoga integrates these mindfulness practices with eleven additional core concepts: empowerment, worth, safety, breath, presence, feeling, grounding, choice, ownership, sustainability, compassion, and self-determination [26]. Metastudies have shown the effectiveness of yoga for reducing trauma symptoms through its promotion of agency, positive self-identity, and a reconnection with one’s body [26]. Based on these concepts, illustrated below is the framework utilized for this intervention [26].

Trauma-informed yoga empowers participants to feel safe while experiencing bodily autonomy, and it allows participants to learn self-acceptance and self-worth through an embodied practice, reintegrating the mind and body [27-28]. This mind–body connection, combined with teaching resilience within the practice, sets trauma-informed yoga apart from regular yoga [28]. Importantly, instructors of a trauma-informed practice have been educated on best practices for working with those who have experienced trauma and can therefore avoid certain triggering postures [26].

Recent studies report that study participants have expressed the ways in which trauma-informed yoga has benefited their lives. Examples include enhancing self-regulation, self-efficacy, promoting an ability to create positive life changes, and even aiding in sobriety [28-29]. These same benefits were found with children who participated in a trauma-informed yoga practice [29]. Student participants reported more fluency in self-expression and identifying emotions, along with an increase in physical health and mental health [29]. Additionally reported benefits went as far as students finding a sense of purpose and a meaning in life [29].

Similar research findings are echoed in educators who participate in a yoga practice. Yoga and mindfulness studies with teachers have shown a correlation between yoga and sleep, wakefulness, and focus [18, 24]. Further, teachers in yoga studies reported spending less time worrying about school outside of work hours and reported being happier with their jobs [18,24]. Interestingly, an increase in heart rate variability (HRV) and a more stable mindset was also found in tandem with a yoga and mindfulness practice [22].

### 3.1. Mindfulness-Based Stress Reduction

As mentioned above, mindfulness-based stress reduction techniques were also integrated into each session in this study. Mindfulness-based stress reduction, or MBSR, is “an established program shown to reduce symptoms of stress, anxiety, and depression.... [and] is believed to alter emotional response by modifying cognitive– affective processes” [30]. According to the American Psychological Association, mindfulness is an “awareness of one’s internal states and surroundings;” this practice can include elements such as breathing techniques, somatic and interoceptive awareness, and meditation [31-32]. Cultivating a practice of mindfulness leads one to redirect negative or destructive cognitive patterns to a non-reactive, non-judgmental observation of one’s thoughts, similarly to cognitive behavioral therapy [31]. Studies illustrate that intentional MBSR practices can improve symptoms of depression, anxiety, and self-esteem in both adults and youth [30]. Therefore, integration of MBSR techniques was complementary to a trauma-informed yoga practice.

### 3.2. Innovation

This project used a novel, trauma-informed yoga intervention in a school setting to promote educator well-being. Few studies have explored teacher wellness interventions for impacts on mental and physiological health, or on classroom behavioral indicators, as is studied here. Delivering the intervention remotely was also a unique aspect of this study, as this modality is just beginning to be studied in mental health interventions due to COVID-19. Finally, this study engaged collaborative partners (a southwestern Montana school district, community yoga studios, and public health organizations—all represented on the study’s Community Advisory Board) in study promotion and recruitment. Given that district administrators requested that the research team focus on educator wellness, this project supported the collaborative approach of CBPR to address community-identified needs.

## 4. Materials and Methods

### 4.1. Research Aim: Pilot a Trauma-Informed Yoga Intervention for Educators in a Southwestern Montana School District

Prior to this study, feedback was gathered from the school district, teachers, and students via informal conversation, open-ended survey responses, and email communications to gauge participant interest in the proposed intervention and to assess the burden from the various assessments. Assessment measures listed below in Table 1 comport with this feedback. Retention and satisfaction of participants, as measured by survey instrumentation from previous pilot studies indicated the need for an ongoing partnership with the school district and an expansion to district teachers for a 6-week intervention. The primary outcomes for teacher wellbeing were career satisfaction and self-efficacy survey scores, with additional secondary measures assessing depressive and anxiety symptomology, sleep quality, resilience, and trauma symptoms. Additional secondary outcomes will include changes in heart rate variability along with cortisol testing to measure physiological changes. Behavioral data from students in teacher participants’ classes was also collected to determine classroom changes for participants. Additionally, all methods and study design were approved by the authors’ university Institutional Review Board prior to the implementation of this project.

### 4.2. Sample and Intervention Design

A recruiting email was sent to all teachers and staff in the school district hosting this study, and participation was incentivized through gift card distribution at the conclusion of the study. Resulting from this recruitment effort, twenty educators volunteered to participate in this study. Our relatively small sample size presented a challenge for this study, as is typical of research held in very rural settings. Professional roles of our participants ranged from classroom and after school teachers to other professional roles within the school setting (such as a school counselor or nurse). Most participants were female (n = 17), and participant ages varied widely (from late 20s to early 70s in age). Ethnically, our sample was homogenous and Caucasian.

Sessions for participants were held twice weekly for six weeks, and each session lasted 45 min via Zoom. Intervention sessions were led by two certified trauma-informed yoga instructors, and the program was held during the first 6 weeks of the third academic quarter. This time period was identified by school district administrators as one that is historically difficult for teachers, as negative student behaviors and absenteeism typically rise during this time. Participants were given the opportunity to choose whether they wanted to attend morning sessions (before school), after school sessions, or to mix and match sessions that worked best with their work schedule. One benefit of a remotely delivered intervention was that it created the opportunity for instructors outside of rural Montana to be a part of the yoga instruction. Thus, one instructor taught virtually from within the rural Montana community, and the other instructor taught remotely from Madison, Wisconsin.

### 4.3. Measures

#### 4.3.1. Adverse Childhood Experience Questionnaire for Adults

Participants completed this questionnaire in order to establish baseline levels of adverse childhood experiences prior to age 18 [33]. This self-report consisted of 10 questions related to the various ACE categories; participants were to count up the total number of events they had experienced in order to determine their baseline ACE score (on a scale of 0–10). A final question asks participants how much they feel these experiences have impacted their health, ranging from “not much”, “some”, and “a lot.” Scores of 0–2 are typically considered to be low ACE scores, 3 as “moderate”, and a score of 4 or higher to be a “high” ACE score. This measure is shown to exhibit a Chronbach’s alpha of 0.88 and a r of 0.71 for validity.

#### 4.3.2. Anxiety

The Generalized Anxiety Disorder Scale (GAD-7) [35] is a 7-item practical self-report anxiety questionnaire where participants are asked how often, during the last 2 weeks, they have been bothered by each of the 7 core symptoms of generalized anxiety disorder. Response options are “not at all”, “several days”, “more than half the days”, and “nearly every day”, scored as 0, 1, 2, and 3, respectively. Therefore, GAD-7 scores range from 0 to 21, with scores of >5, >10, and >15 representing mild, moderate, and severe anxiety symptom levels. In this study, the GAD-7 demonstrated very high internal consistency (Cronbach’s alpha of 0.96) for the sample in this study.

#### 4.3.3. Depressive Symptoms

The Patient Health Questionnaire for Depressive Symptomology for Adolescents (PHQ-A) [39] is a self-report 9-item instrument to assess symptoms of depression among adolescents at study onset. Participants were asked to indicate how often they have been bothered by eight possible problems or symptoms over the last 2 weeks (e.g., “feeling down, depressed, or hopeless”, “feeling tired or having little energy”, and “feeling bad about yourself, or that you are a failure, or have let yourself or your family down”). Each item was rated 0 (not at all), 1 (several days), 2 (more than half the days), or 3 (nearly every day), and items were summed to obtain scale scores. Internal consistency for this scale was high. Using Cronbach’s alpha to measure scale reliability, the PHQ-A scale was 0.90 in the sample.

#### 4.3.4. Resilience

The Connor-Davidson Resilience Scale (CD-RISC) [38] is a self-report 10-item instrument to assess one’s perceptions of self-resilience and agency. Each question is scored on a Likert scale of 0 to 4, where 0 indicates “not at all”, 1 indicates “rarely true”, 2 indicates “sometimes true”, and 4 indicates “true nearly all the time.” Scores can thus range from 0–40, with higher scores indicating higher resilience. Scores are broken into four quartiles; scores from 0–29 exemplify a low resilience score, 30–32 is low-intermediate resilience, 33–36 is high intermediate resilience, and a score of 37–40 exemplifies high resilience. Internal consistency for this study’s scale was high with a Cronbach’s alpha of 0.94 in the sample.

#### 4.3.5. Sleep Disturbances (PROMIS Adult Short Form for Sleep Disturbances)

The PROMISE Adult Short Form for Sleep Disturbances is an 8-item self-reported survey where participants are asked to identify the frequency of sleep disturbances in the previous seven days [36]. Sample questions include “I had difficulty falling asleep” and “I had trouble staying asleep;” responses are recorded on a Likert scale ranging from “Not at all (1)” to “Very Much (5).” Scores can thus range from 8 (indicating high quality sleep) to 40 (indicating very poor-quality sleep). This measure has previously exhibited a Chronbach’s alpha of 0.86 and r validity score ranging from 0.5–0.6. For this study, the Chronbach’s alpha was 0.89.

#### 4.3.6. Adult PTSD Checklist for DSM-5 (PCL-5)

The PCL-5 is a 20-item self-reported measure that assesses the 20 DSM-5 symptoms of post-traumatic stress disorder. The PCL-5 serves a variety of purposes, but for the function of this study, we sought to monitor symptom change during and after the intervention [34]. Questions ask participants how much they were bothered, within the past month, by problems that indicate post-traumatic stress. These items include issues such as “repeated, disturbing dreams of the stressful experience” and having “trouble remembering important parts of the stressful experience.” Each item is scored on a 5-point Likert scale, ranging from 0 (“Not at all”) to 4 (“Extremely”). This instrument has shown strong consistency and reliability, with a Chronbach’s alpha of 0.97, and a validity of r = 0.82. In this study, the internal consistency of items, as measured by Chronbach’s alpha, was also 0.97.

#### 4.3.7. Professional Quality of Life Index (ProQOL)

The Professional Quality of Life Index (ProQOL) is a 30-item quantitative survey instrument that is intended to measure one’s career satisfaction as well as professional burnout. This measure is broken down into three subscale scores rather than a single composite score, including compassion satisfaction, burnout, and secondary traumatic stress [39]. Within each subscale, there are 10 questions related to the category, and responses are recorded on a Likert scale ranging from 1 (“Never”) to 5 (“Very Often”). Within the compassion satisfaction category, a score of 22 or lower indicates low career compassion satisfaction; a score from 23–41 indicates moderate compassion satisfaction, and a score of 42 or more is considered high compassion satisfaction. Both the burnout category and secondary trauma categories utilize the same ranges, with 22 or lower being low burnout/secondary trauma, 23–41 indicating moderate burnout/secondary trauma, and a score of 42 or more showing high burnout/secondary trauma. Internal consistency for this scale was high. Using Cronbach’s alpha to measure scale reliability, the ProQOL scale was 0.94 in the sample.

#### 4.3.8. Teacher Sense of Self-Efficacy Short Form (TSSE)

This 12-item short form survey [40] is intended to measure educators’ senses of self-efficacy and agency in their career. Items include questions like “How much can you do to motivate students who show low interest in school work?” and “How much can you do to control disruptive behavior in the classroom?” Responses are scored on a 9-point Likert scale, ranging from a 1 (“Not at all”) to a 9 (“A Great Deal”). Scores can range from 12, indicating a low sense of self-efficacy as a teacher, to 108, indicating a very high sense of agency as an educator. Again, this scale exhibits strong consistency and reliability with a Chronbach’s alpha of 0.90 and a validity of r = 0.74. Using Cronbach’s alpha to measure scale reliability, the TSSE scale was 0.96 in the sample.

#### 4.3.9. Classroom Behavioral Data

Classroom behavioral data was gathered (from classroom teacher participants) informally through an online survey from participants at the conclusion of each week of the 6-week intervention. Participants were asked to select from a range of frequencies (0, 1–2, 3–5, and 6+) of physical altercations, verbal disruptions, parent contacts, and office referrals within their classrooms each week. These data were quantified descriptively and will be discussed further in the results section.

## 5. Analytical Methods

### 5.1. Survey Analyses

To assess changes pre-and post-intervention, we used descriptive statistics (based upon the individual sum of the scale indicators from each measure for each participant) and also examined differences in means between pre-intervention (T1, during week 1 of the intervention) and post-intervention (T2, at the conclusion of week 6 of the intervention) scores among the teachers who completed the measures at both time points using a paired samples t-test or a Wilcoxon signed rank test with continuity correction for non-normal distributions. These analytical methods enabled us to assess changes in social and emotional functioning of adult participants. All survey measures were disseminated electronically at the same time to all participants.

An a priori power analysis was conducted using G*Power3 [41] to test the difference between two dependent group means (one sample case) using a two-tailed test, a medium-large effect size (d = 0.60), and an alpha of 0.05. Result showed that a total sample of 20 participants was sufficient to achieve a power of 0.80 when relying upon a paired samples t-test, and sufficient for a large effect size (d = 0.90) when relying upon a Wilcoxon signed rank test.

Next, to determine any relationships between individual ACE scores and outcomes from the intervention, analyses of covariance (ANCOVA) following treatment completion was used to evaluate whether there were significant post-treatment differences in teacher-reported measures between participants with low (0–1), moderate (2–3), and high (4+) ACE scores, with pre-intervention teacher-reported symptoms, number of sessions attended, and gender as the covariates. All survey outcome measures were used in these analyses. ANCOVA is the clearest and most straightforward analysis to address each of the analytic goals.

### 5.2. Cortisol Analysis

Study participants were administered salivary cortisol testing at the beginning of weeks 1 and 3, and the conclusion of week 6, in the afternoons at a pre-identified school location. Cortisol was collected at the same time of day (mid-afternoon) during each collection cycle to address the variability of cortisol levels throughout the day. Paired samples t-test assessed differences in means at time 3 vs. time 1, overall and by gender. Specifically, participants provided a saliva sample to PI, which was de-identified (using a code key system) before analysis by the Center for American Indian and Rural Health Equity’s Translational Biomarkers Core Lab at Montana State University. Testing used the Abcam (ab154996) cortisol in vitro competitive ELISA (Enzyme-Linked Immunosorbent Assay) kit designed for accurate quantitative measurement of cortisol in saliva (sensitivity 0.12 ng/mL). Deidentified cortisol data was returned to the PI for re-identification using the code key, and comparisons using paired t tests assessed trends to determine intervention impacts.

### 5.3. HRV Analysis

Heart rate patterns were recorded for 3–5 min using Inner Balance Bluetooth Sensors from HeartMath clipped to each teacher’s ear and sends photoplethysmography (PPG) signals via a wired connection to an accompanying mobile device [42-43]. Data collection occurred in a group setting at a central location within one of the schools of the study school district. Participants downloaded the Inner Balance smartphone application to their personal phones and then paired the Inner Balance Bluetooth Sensors to their personal phones. Once accurate pairing was ensured, participants were instructed to begin a session on their phone application, turn their phone upside down (so as to not follow the HeartMath breathing intervention but merely collect HRV data), and sit quietly for 3–5 min. The research team maintained the timing for each participant’s session. PPG and heart rate variability (HRV) coherence data (calculated by Heartmath EMWavePro software from the PPG data [43] were electronically stored on a password protected HeartMath Cloud Server and with permission from the participant was shared with the research team for analysis using their email and password. All PPG data was de-identified before processing and statistical analysis.

Teacher PPG interbeat interval (IBI) data for pre-, mid-, and post-intervention (time 1, time 2, and time 3) and HRV coherence data were extracted from the HeartMath Cloud Server. We used Mindware HRV Analysis 3.2 to analyze the extracted PPG IBI data to derive mean heart rate and Root Mean Squared of Successive Differences (RMSSD) for a resting baseline of one minute. An automatic artifact detection algorithm was used to detect potential artifacts in the IBI data [44]. Flagged data was visually inspected for artifacts (e.g., identify missed or extra beats) and manually corrected. We calculated RMSSD from the one minute epoch (at least 30 s of continuous artifact-free IBIs was needed) using an interpolation algorithm, linearly detrended to remove non-stationary in the data, and used a Hamming window [45]. Due to the small sample size with complete data across all three time-points, we only tested differences between time 1 and time 3. We conducted a one-tailed, paired sample t-tests (i.e., we expected decreases in heart rate and RMSSD from time 1 to time 3) with time (pre- and post-intervention) as the independent variable and mean heart rate, RMSSD, or HRV coherence as the dependent variable.

## 6. Quantitative Results

### 6.1. Heart Rate Variability

Preliminary examination of the mean heart rate and RMSSD data (n = 21) showed that 2 teachers were missing data at pre-intervention (time 1), 3 teachers were missing data at mid-intervention (time 2), 4 teachers were missing data at post-intervention (time 3), and 1 teacher was missing data across all time points resulting in n = 10 having complete data and n = 14 had data at both time 1 and 3. In addition, inspection of the distribution for each variable showed a single RMSSD outlier at pre-intervention that was greater than 3.29 standard deviations above the mean (i.e., 1614.97). We Winsorized this data point to the next highest value (i.e., 150.42), which resulted in a normal distribution for RMSSD. Result, illustrated below in Table 2, showed no significant change in heart rate from pre- to post-intervention [t(13) = −1.53, p = 0.08], change in RMSSD from pre- to post-intervention [t(13) = 0.88, p = 0.20], or change in HRV coherence from pre- to post-intervention [t(19) = 0.02, p = 0.490].

### 6.2. Cortisol

Salivary cortisol was via passive drool collection and was taken at three time points across the intervention (pre-, mid-, and post-intervention). Paired samples t-test assessed differences in means at time 3 vs. time 1, overall and by gender. There was an unadjusted association between number of treatments (number of classes attended) and cortisol levels at time 3 as compared with time one as shown by an increase in cortisol levels. Cortisol levels were significantly higher at time 3 compared to time 1 (+0.05 mean difference, p = 0.01) Interestingly, male participants’ cortisol levels showed a marked decrease between time 1 and time 2 while female participants’ cortisol levels increased between time 1 and time 2. All genders’ cortisol levels increased between time 2 and time 3.

**Figure 2.**
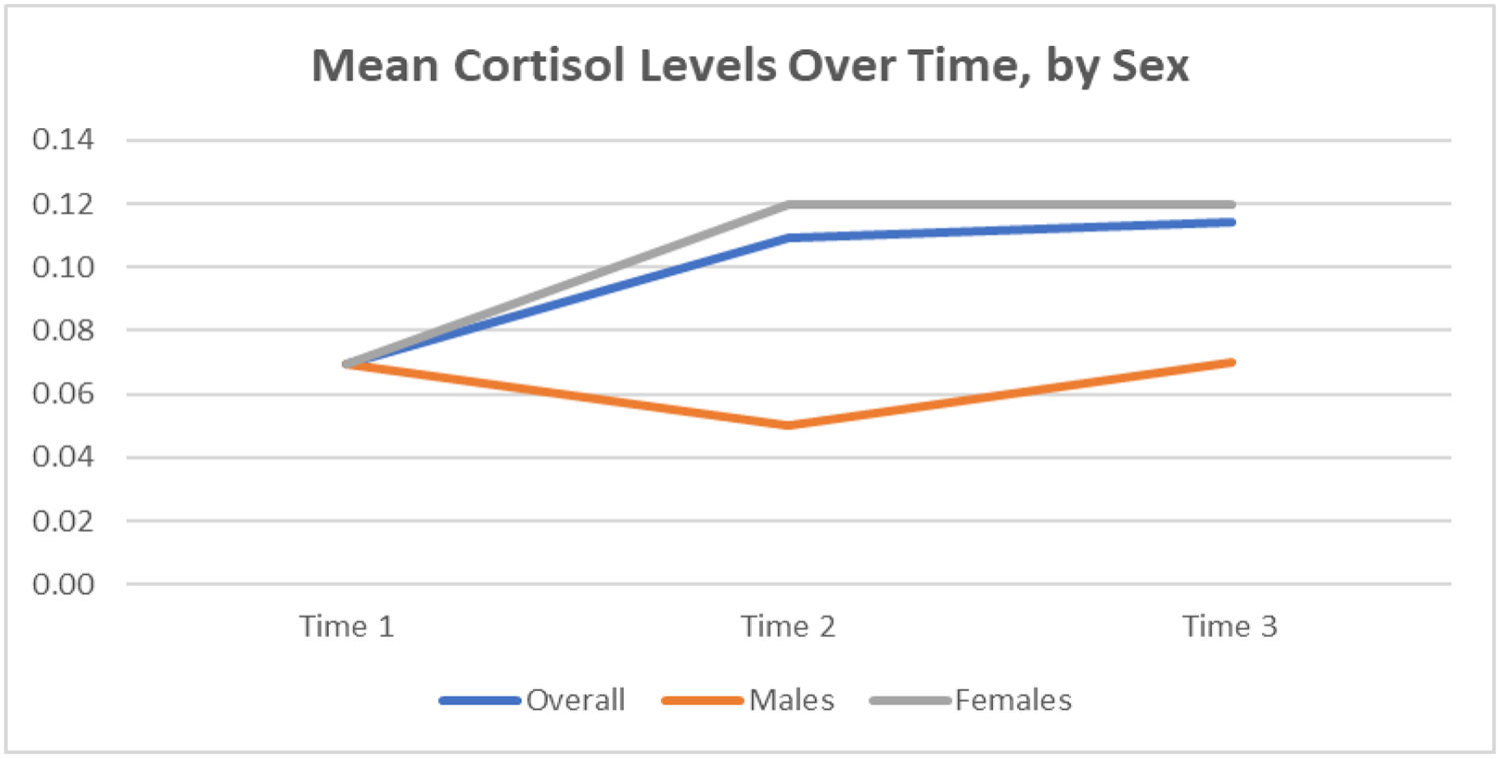
Mean Cortisol Levels Over Time.

When exploring cortisol differences by gender, males in our study appeared to have lower cortisol levels, on average, compared to females. This suggests that sex may serve as an interaction effect when assessing the intervention’s association with cortisol levels. However, with a total sample size of n = 20 (17 females and 3 males), our study is limited in power to detect statistically significant associations by gender. Future research is needed with larger sample sizes and more even distribution of male and female participants to further explore and confirm these findings. Figure 2 below shows the associations of cortisol levels of both the overall treatment group as well as stratified by gender.

**Figure 3.**
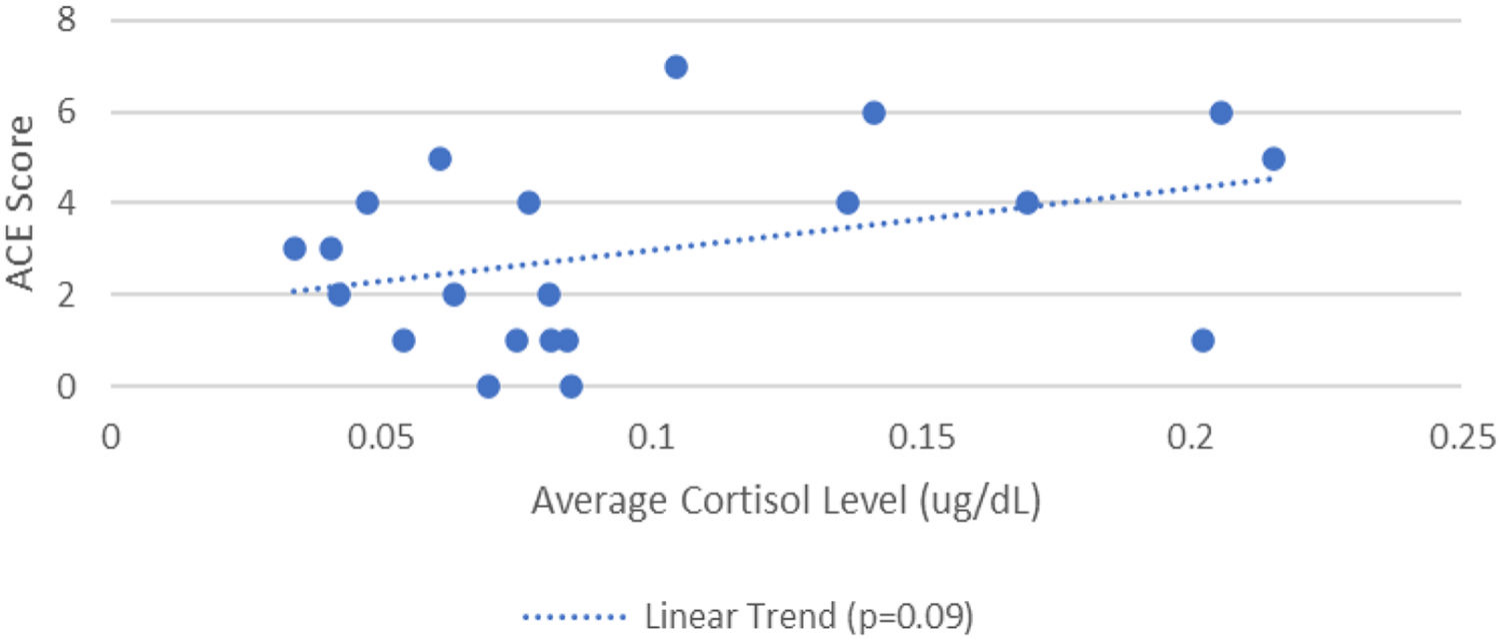
Association Between Cortisol Levels and Adverse Childhood Experience (ACE) Scores.

Following this initial analysis, a linear regression model was then utilized to assess the association between participants’ adverse childhood event (ACE) scores and average cortisol level across the 3 collection timepoints. Results showed an association that bordered statistical significance (p = 0.09) of a 0.01 ug/dl increase in cortisol level, on average, for every 1 unit increase in participants’ ACE scores. Figure 3 below illustrates this association. No other statistically significant results were observed in the cortisol analyses.

**Figure 4.**
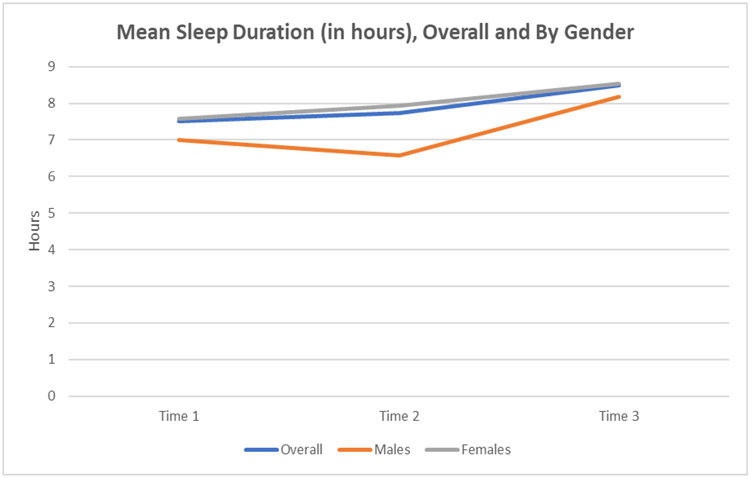
Mean Sleep Duration (in Hours).

During cortisol collection, participants were also asked to self-report sleep duration (collected at time points 1–3). Mean sleep duration (in hours) was examined at each data collection time point, overall and by gender; paired t-tests assessed differences in mean sleep duration at time 3 vs. time 1, again, overall and by gender. Associations between the number of treatments (number of classes attended) and sleep duration (geometric mean change in hours slept per each additional treatment) and sleep quality (odds of “sleeping well” per each additional treatment) were analyzed; these were assessed through an unadjusted association, followed by adjusting for baseline sleep duration/quality and then by baseline sleep duration/quality and gender. Participants’ self-reported sleep duration significantly increased at time 3 compared to time 1 (+0.93 h, on average, p = 0.04). Figure 4 below illustrates these changes across each time point.

### 6.3. Survey Measures

Broadly, there were significant improvements across nearly every validated measure for this study. Table 3 below illustrates the descriptive statistics of these survey measures, and descriptions of statistical results follow.

### 6.4. Depression (PHQ-A)

A paired t-test on the sample of 20 teachers to determine whether there was a statistically significant mean difference between the PHQ-9 score pre and post the intervention indicated that participants had less depression after participating in the study (4.85 ± 3.98) than pre-intervention (8.75 ± 5.67); a statistically significant decrease of 3.90 (95% CI, 1.62 to 6.18), t(19) = 3.58, p < 0.01, d = 0.802

### 6.5. Anxiety (GAD-7)

A paired t-test indicated that there was a statistically significant mean difference between the GAD-7 score pre and post the intervention. Teachers had less anxiety after participating in the study (4.85 ± 3.99) than prior-to participation (13.9 ± 6.63); a statistically significant decrease of 9.05 (95% CI, 6.64 to 11.46), t(19) = 7.87, p < 0.001, d = 1.76.

### 6.6. Resilience (CD-RISC)

Again, using a paired t-test, analyses indicated that there was a statistically significant mean difference in CD-RISC before and after the yoga intervention. Teachers had statistically significant more resilience after participating in the study with a difference of 10.15 (95% CI, −11.86 to −8.44), t(19) = −12.45, p < 0.001, d = −2.78. Mean scores post-intervention (38.5 ± 7.12) were considerably higher than the average pre-score (28.35 ± 6.00).

### 6.7. Trauma Symptoms (PCL-5)

An additional paired t-test indicated that there was a statistically significant mean difference between the PCL score pre and post the intervention. Teachers had less PTSD after participating in the study (14.75 ± 13.46) than prior-to study participation (26 ± 20.74); a statistically significant decrease of 11.25 (95% CI, 5.58 to 16.92), t(19) = 4.16, p < 0.001, d = 0.93.

### 6.8. Teacher Sense of Self Efficacy Scales (TSSE)

Due to a severe lack of normality in the self-efficacy scores, a Wilcoxon Signed Rank Test was performed to determine if there was a statistically significant difference in overall self-reported mean self-efficacy before and after participating in the intervention. A total of 20 participants were used in the analysis. The test revealed that there was a statistically significant difference in mean total self-efficacy score between the two groups (z = −2.774 p = 0.0055). These results indicate that the yoga intervention had a significant effect on the increasing teacher self-efficacy.

Further, a paired t-test indicated that there was a statistically significant mean difference between the instructional practices score pre and post the intervention. Teachers reported higher self-efficacy when referring to their instructional practices after participating in the study (29.0 ± 3.51) than prior-to study participation (25.6 ± 6.97; a statistically significant difference of −3.4 (95% CI, −6.02 to −0.78), t(19) = −2.71 p < 0.05, d = −0.61. However, there were neither significant differences on the classroom management subscale (t(19) = −1.93, p = 0.07) nor the student engagement scale (t(19) = −1.45, p = 0.16)

### 6.9. Sleep Disturbances (PROMIS Sleep Scales)

A paired t-test on the sample of 20 teachers to determine whether there was a statistically significant mean difference between sleep quality pre and post the intervention indicated that participants had lower PROMIS scores after participating in the study (20.1 ± 6.46) than pre-intervention (23.4 ± 2.82); a statistically significant decrease of 3.3 (95% CI, 0.15 to 6.45), t(19) = 2.20, p < 0.05, d = 0.49.

### 6.10. Professional Quality of Life Index (ProQOL)

The only subscale on the ProQOL measure that was statistically significant was burnout. A paired t-test indicated that there was a statistically significant mean difference between the burnout score pre and post the intervention. Teachers reported less burnout after participating in the study (23.39 ± 7.32) than prior-to study participation (34.4 ± 4.43); a statistically significant decrease of 11.2 (95% CI, 8.13 to 14.27), t(19) = 7.63, p < 0.001, d = 1.71. There were no statistically significant differences in compassion satisfaction (t(19) = −1.28, p = 0.22) or secondary traumatic stress among teachers between pre and post-intervention (t(19) = 0.96, p = 0.35. Table 4 below indicates pre- and post-intervention differences in outcomes for each measure.

### 6.11. Variation by Number of ACES

In this sample, 35% of participants reported one or fewer ACES, 25% reported a moderate number and 40% reported a high number of ACES. Across outcomes, the ANCOVAS indicated that the post-test means, adjusted for pre-test scores, gender, and number of sessions attended, did not significantly differ between the three ACE groups of low (0–1), moderate (2–3), and high (4+) for any of the measures in the study, which included anxiety (GAD-7), depression (PHQ-A), resilience (CD-RISC), adult PTSD (PCL-5), sleep quality (PROMIS), self-efficacy and its three subscales of instructional practice, classroom management, and student engagement, and the three professional quality of life indices (PROQOL).

### 6.12. Classroom Behavioral Data

Baseline, teacher-reported negative classroom behaviors during the initial implementation of the study showed most teachers had high rates of verbal disruptions and moderate numbers of physical altercations, negative parental contacts, and office referrals. Weeks two through three showed a reduction in higher frequencies (6+ occurrences) across all four categories. Weeks four and five corresponded with an increase in occurrences, which may be attributed to a combination of negative events that occurred within the school district during that time. However, in week six, negative classroom behaviors generally decreased in the highest frequency category (6+ occurrences), followed by an increase in moderate frequencies (1–5 occurrences). Across the six-week intervention period, the greatest reduction in negative classroom behaviors was in the verbal disruption category.

## 7. Discussion

### 7.1. HRV and Cortisol Analyses

Our HRV analyses found no statistically significant changes resulting from this intervention. However, it is interesting to note that cortisol levels increased from mid-intervention to post-intervention. With our cortisol analysis, one outlier indicated that they felt ill during the third collection; however, our results did not change when we removed this outlier in a sensitivity analysis. The authors of this study have several hypotheses regarding these unexpected physiological findings between each data collection time point.

First, cortisol levels of participants fell within a normal and healthy range at each time point, so it is possible that this intervention has less impact when baseline cortisol levels are already quite low (when comparing them to previous studies involving this intervention and teenagers) [46-47]. Supporting this hypothesis, Galvan notes “previous work has shown that, under identical stress conditions, teens show greater cortisol release than adults” [48]. Cahn et al. [49] note additional research further indicates that increases in cortisol following a yoga intervention may be a normal finding for adults, especially when combining yoga and mindfulness practices [50-54]. Moreover, a comprehensive literature review of yoga and heart rate variability research also suggests that yoga interventions do increase heart rate variability among participants [55].

Finally, and perhaps most importantly, there were two crises that occurred within the school system during week 5 that the research team feels strongly impacted the physiological results of this study. In this particular district, masking for COVID-19 was a hotly contested controversy, and in week 5, masking became optional, leading to many angry parents disrupting classrooms and schools that week. Additionally, there was a severe negative event involving student and school safety that undoubtedly greatly increased participants’ stress levels (due to confidentiality, the research team is unable to disclose the specifics of this severe event).

### 7.2. Survey Outcomes

Survey results were overwhelmingly positive with statistically improved mental health and career satisfaction/self-efficacy outcomes in nearly every measure. This indicates that a trauma-informed yoga intervention can not only improve educator wellbeing, but it also holds the potential to reduce career burnout while improving a sense of self-efficacy in teaching.

Curiously, when examining educators’ self-reported ACE scores across outcomes, there was no relationship between ACE levels and survey results. Thus, this intervention was as effective for those with low ACE scores as those with moderate or high ACE scores. In previous iterations of this study where high school students participated in this intervention, similar results were reported with ACE scores and mental health outcomes [46]. The fact that this intervention is beneficial for all levels of childhood trauma for both students and educators highlights its potential as being advantageous for all students and educators, regardless of ACE scores.

## 8. Study Limitations

There were several limitations in this study. First and foremost, our limited sample size resulted in limited statistical power. In rural settings, such as these, sample size can often be one of the primary barriers to research participation, as there is simply a lack of available and/or interested participants. Additionally, our sample was fairly heterogeneous in terms of educator roles; our participants ranged from after school teachers to classroom teachers and from resource teachers to a school nurse. Ethnically, however, our sample was homogenous (though diverse in age range) and was composed of mostly white women.

Additionally, our yoga instructors also introduced another element of variability. While each teacher was female and trauma-certified, each teacher very much had her own unique “style” of teaching. One teacher was only available in the mornings, and one teacher was available only in the evenings; therefore, some participants only had access to one, rather than both, styles of teaching, leading to some inevitable variations in the intervention delivered. However, many participants noted that they liked having the access to both teachers, as they were allowed to “mix and match” sessions according to their own scheduling availability.

Finally, there were multiple technological challenges when attempting to gather participant heart rate variability. The Inner Balance ear sensors frequently disconnected via Bluetooth from participants’ smartphones, application sessions inexplicably ended prematurely, and participants often did not adhere to protocols during data collection times (i.e., talking and moving around while the data collection was in process). While these limitations exist, they are also indicative of the nature of applied research in school settings, which requires agility, flexibility, and unexpected outcomes, especially when working with rural populations.

## 9. Conclusions

In an anonymous survey given to teachers in this school district, prior to the inception of this study, teachers were asked how they were coping with job-related stress since COVID-19; one teacher reported, “I go home and drink”. Teacher mental health has historically been a concern nationally, as well as in rural Montana, due to high stress levels associated with the profession [14,16,21]. Since the onset of the COVID-19 pandemic, mental health crises are on the rise for both adolescents and adults [56-58]. Moreover, adults reporting symptoms of an anxiety disorder quadrupled from June 2019 to December 2020 [59].

Teachers experiencing the collective trauma and associated stressors of COVID-19 are particularly vulnerable to poor mental health outcomes, including professional burnout characterized by anxiety and emotional exhaustion [20]. Importantly, when teachers are dysregulated, their students are more stressed and have lower academic achievement [60]; conversely, improvements in teacher wellbeing and reductions in depressive symptomology are associated with improvements in student wellbeing and psychological difficulties [61]. Thus, given the enormous pressures of this profession, combined with the collective trauma of a global pandemic, the mental health and well-being of teachers has never been so critical–both for the benefit of educators as well as their students.

## Figures and Tables

**Figure 1.**
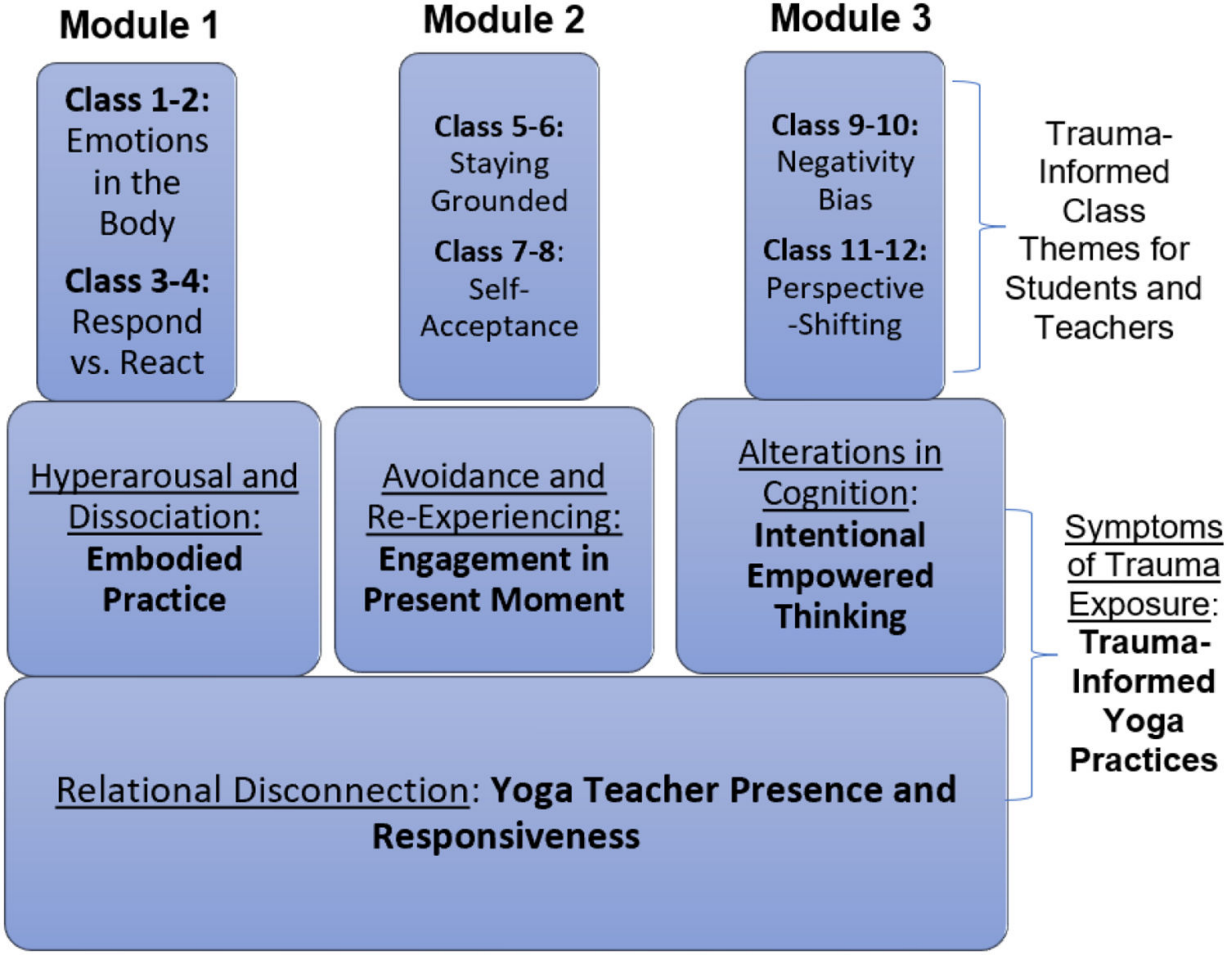
Conceptual Framework for Trauma-Informed Yoga Intervention, adapted from Cook-Cottone et al., 2017.

**Table 1.** Assessment Measures.

Description of Measure	Source	Timeline for Collection	Cronbach’s Alpha/Validity & Reliability
Adverse Childhood Experiences Questionnaire for Adults	[33]	Pre-intervention (to determine ACE scores of participants)	Chronbach’s alpha: 0.88 Reliability: 0.71
PTSD Checklist for DSM-5 (PCL-5)	[34]	Pre- and post-intervention	Chronbach’s alpha: 0.97 Reliability: 0.82
Generalized Anxiety Disorder Scale (GAD-7)	[35]	Pre- and post-intervention	Cronbach’s alpha: 0.79–0.91 Reliability = 0.85
PROMIS Adult Short Form for Sleep Disturbances	[36]	Pre- and post-intervention	Chronbach’s alpha: 0.86 Validity: r = 0.05–0.6
Patient Health Questionnaire for Depressive Symptomology (PHQ-9)	[37]	Pre- and post-intervention	Chronbach’s alpha = 0.835 Reliability = 0.875 Validity = 89.5%
Connor-Davidson Resilience Scale (CD-RISC)	[38]	Pre- and post-intervention	Chronbach’s alpha = 0.94 Reliability = 0.96
Professional Quality of Life Index (Pro-QOL)	[39]	Pre- and post-intervention	Cronbach’s alpha: 0.90 Reliability = 0.80–0.90
Teachers’ Sense of Self-Efficacy (Short Form)	[40]	Pre- and post-intervention	Cronbach’s alpha: 0.90 Reliability = 0.74
Cortisol salivary assays	Salimetrics Laboratories	Pre (beginning of week 1)-, mid- (week 3) and post-intervention (end of week 6)	Mean accuracy of salivary cortisol testing > 90%
Heart rate variability data	HeartMath Institute (Em Wave Pro Plus Software and the HeartMath Inner Balance PPG sensor)	Pre- (1 week prior to intervention to establish a baseline), mid- (week 3) and post-intervention (at conclusion of week 6)	Pearson correlation between electrocardiogram (ECG) and ear clip pulse plethysmograph (PPG) device (Em Wave Pro Plus) mean resting baseline = 0.997, *p* < 0.01; RMSSD Pearson correlation = 0.958, *p* < 0.01

**Table 2.** Mean Heart Rate, RMSSD, and HRV Coherence for Pre- and Post-Intervention.

	Pre-Intervention	Post-Intervention
Mean Heart Rate	72.32 (09.49)	75.56 (13.71)
RMSSD	66.88 (35.57)	59.36 (29.79)
HRV Coherence	01.45 (00.62)	01.45 (00.54)

Note: RMSSD = Root Mean Squared of Successive Differences; HRV = Heart Rate Variability. Means reported in Table 2 are from available data for each variable (*n* = 14 for mean heart rate and RMSSD; *n* = 20 for HRV Coherence) at pre- and post-intervention.

**Table 3.** Descriptive Statistics of Survey Measures, Pre and Post.

	Pre		Post	
	Mean	St. Dev	Mean	Std. Dev
PHQ-A	8.75	5.674	4.85	3.977
GAD7	13.9	6.632	4.85	3.99
CD-RISC	28.35	6.002	38.5	7.119
PLC5	26	20.736	14.75	13.463
PROMIS	23.4	2.817	20.1	6.456
Self-Efficacy (overall)	74.85	20.306	82.3	8.694
Self-Efficacy: Student Engagement	23.25	6.624	24.9	4.217
Self-Efficacy: Instructional Practice	25.6	6.969	29	3.509
Self-Efficacy Classroom Management	26	7.233	28.4	3.016
PROQOL: Compassion Satisfaction	37.95	7.437	39.2	6.55
PROQOL: Burnout	34.4	4.43	23.2	7.324
PROQOL: Secondary Traumatic Stress	23.9	8.723	22.55	6.411

**Table 4.** Pre/Post Difference for Outcomes.

Outcome ^a^	Difference	95% CI	*p*	Cohen’s d
PHQ-A	3.9	1.62, 6.17	0.002	0.80
GAD-7	9.05	6.64, 11.46	0.000	1.76
CD-RISC	10.15	11.86, 8.44	0.000	2.78
PCL-5	11.25	5.58, 16.92	0.0005	0.93
Self-Efficacy	−7.45	−9.0,−2.0	0.006	−0.50
Self-Efficacy: Instructional Practice	−3.4	−6.02, −0.78	0.014	−0.61
Self-Efficacy: Classroom Management	−2.4	−5.00, 0.20	0.069	−0.43
Self-Efficacy: Student Engagement	−1.65	−4.03, 0.73	0.164	−0.32
PROMIS	3.3	0.15, 6.45	0.041	0.49
ProQOL: Burnout	11.2	8.13, 14.27	0.000	1.71
ProQOL: Compassion Satisfaction	−1.25	−3.29, 0.79	0.216	−0.29
ProQOL: Secondary Traumatic Stress	1.35	−1.59, 4.29	0.348	0.22

a Wilcoxon signed rank test with continuity correction.

## Data Availability

Due to the nature of this research, participants of this study did not agree for their data to be shared publicly, so supporting data are not available.
